# Evasion strategies of porcine reproductive and respiratory syndrome virus

**DOI:** 10.3389/fmicb.2023.1140449

**Published:** 2023-03-17

**Authors:** Xin-xin Chen, Songlin Qiao, Rui Li, Jing Wang, Xuewu Li, Gaiping Zhang

**Affiliations:** ^1^Key Laboratory of Animal Immunology of the Ministry of Agriculture, Henan Provincial Key Laboratory of Animal Immunology, Henan Academy of Agricultural Sciences, Zhengzhou, Henan, China; ^2^Jiangsu Co-Innovation Center for Prevention and Control of Important Animal Infectious Diseases and Zoonoses, Yangzhou University, Yangzhou, Jiangsu, China

**Keywords:** immune evasion, porcine reproductive and respiratory syndrome virus (PRRSV), innate immunity, adaptive immunity, miRNA

## Abstract

During the co-evolution of viruses and their hosts, viruses have developed various strategies for overcoming host immunological defenses so that they can proliferate efficiently. Porcine reproductive and respiratory syndrome virus (PRRSV), a significant virus to the swine industry across the world, typically establishes prolonged infection *via* diverse and complicated mechanisms, which is one of the biggest obstacles for controlling the associated disease, porcine reproductive and respiratory syndrome (PRRS). In this review, we summarize the latest research on how PRRSV circumvents host antiviral responses from both the innate and adaptive immune systems and how this virus utilizes other evasion mechanisms, such as the manipulation of host apoptosis and microRNA. A thorough understanding of the exact mechanisms of PRRSV immune evasion will help with the development of novel antiviral strategies against PRRSV.

## 1. Introduction

Porcine reproductive and respiratory syndrome (PRRS), characterized by respiratory distress in pigs of all ages and reproductive failures in sows, is currently prevalent in all pig farming countries, and it has imposed a huge economic burden on the global pig industry every year since its emergence in the 1980s (Holtkamp et al., 2013). The causative agent of PRRS is PRRS virus (PRRSV), which is an enveloped, single-stranded, positive-sense RNA [(+) ssRNA] virus that has recently been reclassified into the genus *Betaarterivirus*, family *Arteriviridae*, order *Nidovirales*.^1^ PRRSV is grouped into two genotypes: PRRSV-1, or the European genotype (prototype Lelystad), and PRRSV-2, or the North American genotype (prototype VR-2332). PRRSV strains are characterized by extensive genetic and antigenic variation, and they recombine frequently, leading to the emergence of diverse novel strains (Wang et al., 2017; Yu et al., 2020, 2022; Sun et al., 2022). For example, the highly pathogenic PRRSV (HP-PRRSV) strain, with a 30-amino-acid (aa) deletion in non-structural protein 2 (nsp2), became endemic following a sudden outbreak in China in 2006 (Tian et al., 2007), and NADC30-like strains, characterized by a unique discontinuous 131-aa deletion in the nsp2-coding region, preferentially recombine with other PRRSV strains. This contributes to the complexity of PRRSV epidemiology, prevention, and control (Zhao et al., 2015; Zhou et al., 2015; Wang et al., 2018).

The PRRSV genome, approximately 15.4 kb in length with a 5’-cap and 3’-polyadenylated [3’-poly (A)] tail, consists of at least 11 open reading frames (ORFs): ORF1a, ORF1b, ORF2a, ORF2b, ORFs3–7, ORF5a, and ORF2TF (Fang et al., 2012). ORF1a and ORF1b occupy approximately 75% of the 5’-end of the viral genome and encode two large polyproteins, pp1a and pp1ab, which are processed into at least 16 non-structural proteins (nsps): nsp1α, nsp1β, nsp2–6, nsp2TF, nsp2N, nsp7α, nsp7β, and nsp8–12. nsp2TF and nsp2N are expressed through a –2/–1 programmed ribosomal frameshift mechanism (Fang et al., 2012; Li et al., 2019). ORF2a, ORF2b, ORFs3–7, and ORF5a occupy the 3’-end of the genome and encode glycoprotein (GP) 2, envelope (E), GP3, GP4, GP5, ORF5a, membrane (M), and nucleocapsid (N) proteins (Fang and Snijder, 2010).

Porcine reproductive and respiratory syndrome virus infection is characterized by a poor induction of the host innate immune response along with delayed protective antibody and cell-mediated immune responses, which make PRRS difficult to prevent, control, and eliminate (Lunney et al., 2016). PRRSV can cause prolonged infection in pigs for long periods of time, up to 251 days (Beyer et al., 2000; Wills et al., 2003). Thus, a comprehensive understanding of how PRRSV evades host antiviral responses will be critical to the development of novel therapies for the prevention and control of PRRS. Here, we present an overview of the host evasion strategies that are employed by PRRSV.

## 2. Escape from host innate immune responses

### 2.1. Host innate immune responses against PRRSV

The innate immune system is the first line of host defense against pathogens. Once a virus infection occurs, activation of the host innate immune system is needed to establish an antiviral state that can limit viral spread and shape the subsequent adaptive immune response (Blasius and Beutler, 2010). The host innate immune response is initiated by pattern recognition receptors (PRRs) that act as sensors to detect conserved components of pathogens, termed pathogen-associated molecular patterns (PAMPs), and then trigger a series of downstream pathways that induce the production of type I interferons (IFNs: IFN-α/β), IFN-stimulated genes (ISGs), and various inflammatory cytokines and chemokines (Takaoka and Yanai, 2006; Koyama et al., 2008).

Pathogen-associated molecular patterns are unique and conserved features present in pathogens but absent from host cells (e.g., nucleic acid sequences or RNA secondary structures) that allow a cell to distinguish self from non-self and thus activate host immune responses against the pathogens. For RNA viruses, protein and nucleic acid products of infection or replication can serve as viral PAMPs that are detected by host PRRs (Goubau et al., 2013). Host PRRs are divided into several families: Toll-like receptors (TLRs), RIG-I-like receptors (RLRs), NOD-like receptors (NLRs), C-type lectin receptors (CLRs), AIM2-like receptors (ALRs), and cyclic GMP-AMP synthase (cGAS) (Blasius and Beutler, 2010; Takeuchi and Akira, 2010; Chen and Jiang, 2013). The main PRRs for sensing RNA viruses are the TLRs (mainly TLR3 and endosomal TLR7–8) and cytosolic RLRs (RIG-I, MDA5, NLRP3, and NOD2) (Kato et al., 2006; Goubau et al., 2013). TLR3 recognizes double-stranded (ds) RNA or the intermediate RNAs generated during the replication of different viruses. TLR7 (or TLR8 in humans) recognizes ssRNA (Fitzgerald and Kagan, 2020). RIG-I recognizes relatively short duplexed regions of RNA with blunt-ended 5’-triphosphate or 5’-diphosphate, which are often present at the end of the genomic RNAs of (+) ssRNA viruses (Hornung et al., 2006; Goubau et al., 2014). Viral RNA containing complex secondary structures that can form short double-stranded structures with perfectly blunt ends or contain an A/U-rich motif in the 5’/3’ untranslated region (UTR), such as those reported in hepatitis C virus (HCV) and human immunodeficiency virus (HIV), can still be detected by RIG-I (Saito et al., 2008; Solis et al., 2011). MDA5 preferentially binds to long dsRNA (>1,000 bp) with no end specificity (Reikine et al., 2014). MDA5 is uniquely triggered during picornaviridae, caliciviridae, and coronaviridae infections or in the presence of synthetic RNA polymers consisting of poly (I:C) (Kato et al., 2006; Deddouche et al., 2014).

#### 2.1.1. Immune sensing of PRRSV by the host

The mechanism of PRRSV recognition by the host remains incompletely understood. The research on TLR mainly focuses on the regulation of TLR expression upon PRRSV infection. A previous study indicated that the infection of pigs with PRRSV tends to increase the expression of TLR 2, 3, 4, 7, and 8 mRNAs in at least one of the lymphoid tissues or cells (Liu et al., 2009). Inhibition of TLR3, 4, 7 were observed in PAMs or/both immature DCs at 6 h post-infection (hpi), and abolished at 24 hpi (Miller et al., 2009; Chaung et al., 2010). These results might depend on the viral strain and in a timely manner (Kuzemtseva et al., 2014; Li and Mateu, 2021). Porcine TLR3 and TLR7 detect double stranded RNA (dsRNA) and single stranded RNA (ssRNA), respectively. But evidence for a direct interaction with PRRSV is lacking (Sang et al., 2008; Liu et al., 2009). In a recent study, DDX19A was identified as a novel cytosolic RNA sensor that binds PRRSV RNA to activate the NLRP3 inflammasome, resulting in interleukin (IL)-1β production (Li et al., 2015). Our previous studies found that the 3’ UTR pseudoknot region of PRRSV could act as a PAMP recognized by RIG-I and TLR3 to induce the production of type I IFNs (Xie et al., 2018).

### 2.2. Evasion of the innate immune response

**FIGURE 1:**
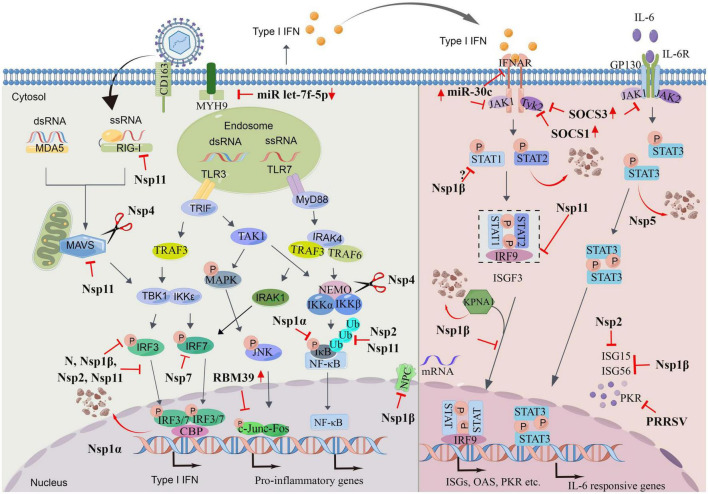
Porcine reproductive and respiratory syndrome virus (PRRSV) evasion strategies. PRRSV utilize numerous mechanisms to evade host antiviral immune responses. The figure depicts the actions of viral proteins upon different molecules involved interferons (IFN) and pro-inflammatory genes producing signaling pathways and IFN- Janus kinase/signal transducers and activators of transcription (JAK/STAT)-IFN-stimulated genes (ISGs) pathways. The red arrow indicates that PRRSV induces upregulation/downregulation of the protein. The scissor indicates the cleavage role of indicated protein. P, phosphate; Ub, ubiquitin. Drawn by Figdraw.

Porcine reproductive and respiratory syndrome virus shows strict tropism for cells from the monocyte/macrophage lineage (Van Breedam et al., 2010). Infection of porcine alveolar macrophages (PAMs) with PRRSV significantly reduced the ability of these cells to respond to TLR3 ligation (Miller et al., 2009). Preliminary studies showed that PRRSV-infected PAMs exhibit delayed and low-level type I IFN activity. Levels of IFN-α are low in PRRSV-infected pigs and are not detectable in the lungs of PRRSV-infected pigs where this virus replicates prolifically (Miller et al., 2009; Kittiwan et al., 2019). PRRSV was also reported to repress type I IFN and proinflammatory cytokine responses in porcine plasmacytoid dendritic cells (pDCs), which are a major source of IFN-α and other inflammatory cytokines with a rapid reaction to virus infection (Calzada-Nova et al., 2011). However, the inhibition in pDCs was demonstrated to be strain-dependent and mostly restricted to PRRSV-2 (Baumann et al., 2013). Further research shows that, to maintain viral replication and spread, PRRSV has evolved strategies for hindering innate immune signaling; PRRSV can target any step in this process, e.g., evading PRR detection, targeting the activation of intermediate adaptors and kinases, or disrupting transcription factors and ultimately inhibiting the production of IFN, activation of ISGs and antiviral restriction factors. PRRSV is capable of repressing an elicited IFN-α response, as well as the responses of some cytokines, such as IL-2, IL-6, and tumor necrosis factor alpha (TNF-α) (Calzada-Nova et al., 2011). Several nsps and structural proteins have been identified as playing roles in the suppression of the innate immune response through different mechanisms, which have been widely reviewed (Yoo et al., 2010; Huang et al., 2015; Wang et al., 2021). Therefore, this review provides only a brief overview on this aspect of innate immune suppression by PRRSV (Figure 1).

#### 2.2.1. Targeting the IFN-producing signaling pathway

Firstly, PRRSV can evade sensing by PRRs. Arteriviruses have been reported to induce the formation of double-membrane vesicles (DMVs) carrying the viral replication complex; these DMVs might help to hide the viral RNAs from cellular detection, thus delaying IFN production (Spilman et al., 2009; Knoops et al., 2012). Secondly, PRRSV targets the activation of intermediate adaptors and kinases. For example, nsp4, a 3C-like proteinase, was shown to mediate nuclear factor-κB (NF-κB) essential modulator (NEMO) and VISA cleavage to suppress type I IFN induction (Chen et al., 2014; Huang et al., 2014, 2016). PRRSV infection inhibits the production of IFN-β by interfering with the activation of IPS-1 in the RIG-I signaling pathway or by reducing the expression of MAVS mRNA *via* nsp11, in a manner dependent on its endoribonuclease activity (Luo et al., 2008; Sun et al., 2016). Finally, PRRSV also can target transcriptional factors directly or indirectly, such as NF-κB, IFN-regulatory factor (IRF) 3 (IRF3), and IRF7, which are also one of the most common innate immune escape strategies of viruses. For example, nsp1α inhibits IκB phosphorylation and blocks NF-κB translocation to the nucleus, leading to the inhibition of NF-κB-stimulated genes expression (Song et al., 2010). Nsp1β inhibits IRF3 phosphorylation and nuclear translocation (Beura et al., 2010; Sagong and Lee, 2011). RNA-binding protein 39 (RBM39), which is upregulated by PRRSV, prompts PRRSV proliferation *via* altering c-Jun phosphorylation, nucleocytoplasmic translocation, and stabilizing and binding with viral RNA (Song et al., 2021).

#### 2.2.2. Targeting of Janus kinase/signal transducers and activators of transcription (JAK/STAT) signaling pathways

Interferon-stimulated genes can directly antagonize the PRRSV lifecycle. Cytokines such as Type I IFNs induce the expression of ISGs by activating the phosphorylation of both STAT1 and STAT2, which form heterotrimers with IRF9 and translocate to the nucleus. PRRSV was reported to inhibit IFN–JAK/STAT signaling *via* blocking the nuclear translocation of STAT1/STAT2/IRF9 heterotrimers, also known as ISG factor 3 (ISGF3), thereby inhibiting the production of ISG15 and ISG56 (Chen et al., 2010; Patel et al., 2010; Wang et al., 2019). Further studies showed that nsp1β mediates the nuclear import of ISGF3 by inducing the degradation of karyopherin-α1 (KPNA1), the karyopherin for ISGF3 nuclear translocation (Wang et al., 2013b). However, whether nsp1β affects STAT1 phosphorylation is controversial and more research is needed (Chen et al., 2010; Patel et al., 2010). Nsp11 antagonizes IFN signaling *via* mediating STAT2 degradation (Yang et al., 2019). The N protein was found to inhibit the activity of IFNs, possibly through blocking STAT1 nuclear translocation (Wang et al., 2013a). STAT3, a pleiotropic signaling mediator of many cytokines, is involved in multiple cellular processes and host immune responses (Shuai and Liu, 2003). PRRSV nsp5 antagonizes JAK/STAT3 signaling by inducing the degradation of STAT3 *via* the ubiquitin–proteasomal pathway (Yang et al., 2017).

#### 2.2.3. Targeting ISGs or other antiviral proteins

Porcine reproductive and respiratory syndrome virus also moderate the antiviral immunity through manipulating the production or function of ISGs or other antiviral proteins. For instance, porcine mRNA-decapping enzyme 1a (DCP1a) is an ISG induced by IFN-α that participates in removing the 5’-methylguanosine cap from eukaryotic mRNA. PRRSV nsp4 is responsible for cleaving DCP1a to impair its antiviral activity (Tao et al., 2018). Cholesterol-25-hydroxylase (CH25H), a conserved ISG-encoded polytopic membrane protein, has been reported to broadly inhibit the growth of many viruses (Liu et al., 2013). Nsp1β and nsp11 antagonize the antiviral activity of CH25H *via* lysosomal degradation (Dong et al., 2018). Protein kinase R (PKR) is an ISG whose activity is triggered by the presence of foreign dsRNA. PKR prevents virus replication by inhibiting the translation of viral mRNAs through phosphorylation of eIF2a, which is responsible for the initiation of polypeptide synthesis (Medina et al., 2020). However, PRRSV inhibits PKR activation and the PKR-mediated phosphorylation of eIF2a at the early stage after inoculation (Xiao Y. et al., 2016). Nsp1β is a stress-responsive protein, enters virus-induced stress granules (SGs) during infection, and repurposes SGs into a proviral platform, where it co-opts the SG core component G3BP1 to interact with PKR in a regulated manner (Gao et al., 2022). The latest research shows that PRRSV modulates mucosa-associated lymphoid tissue lymphoma translocation protein 1 (MALT1) expression to antagonize anti-PRRSV RNases N4BP1 and monocyte chemotactic protein-induced protein (MCPIP1) upon infection, thereby facilitating viral replication (Gu et al., 2022). PRRSV also significantly upregulates MCPIP1 expression in lungs of PRRSV-infected piglets, as well as in cells cultured *in vitro* to promote replication in the early stage of virus infection (Zheng et al., 2021). MCPIP1, is a broad-spectrum host antiviral protein that maintains low expression levels in most cell types and is induced by many inflammatory genes.

#### 2.2.4. Dysregulation of natural killer (NK) cell function

Natural killer (NK) cells, an important cell population of the innate immune system, have the abilities to identify infected or transformed cells, elicit cytotoxicity, and produce immune-regulatory cytokines (Gerner et al., 2009). Pigs have relatively more NK cells than other animals and humans (Gerner et al., 2009). However, previous studies indicated that the NK-cell cytotoxicity against PRRSV-infected PAMs was suppressed from 6 to 12 h post-infection (Cao et al., 2013). Consistently, a significant reduction in NK-cell-mediated cytotoxic function in PRRSV-infected pigs was detected (Renukaradhya et al., 2010). But the insufficient NK-cell activity during PRRSV infection is not only because of the insufficient activation of NK cells (Cao et al., 2013). At present, there has been little research on this topic, and the strategies adopted by PRRSV-infected PAMs to resist NK-cell cytotoxicity are still unknown. Further studies are urgently needed to determine the underlying mechanisms.

Despite the many studies that have been conducted on PRRSV evasion of innate immunity, more research is needed to reveal the details of the underlying mechanisms because most of the previous work has lacked evidence of direct or indirect interaction.

## 3. Escape from adaptive immunity

The adaptive immune response is essential for the development of protective immunity against pathogen infection. However, the suppressed innate immune response in early phase of PRRSV infection can potentially lead to the generation of poor adaptive immune responses, such as abnormal B-cell and T-lymphocyte proliferation, delayed neutralizing antibody responses, and poor induction of PRRSV-specific IFN-γ-producing cells (Wongyanin et al., 2012). In addition to affecting adaptive immunity *via* its suppression of innate immunity, PRRSV can also directly target adaptive immunity for evasion.

### 3.1. Impairing antigen presentation

Dendritic cells (DCs) are potent antigen-presenting cells that deliver differentiation signals to T cells. Thus, the impairment of DC function by viruses has been proposed as a mechanism for viral persistence and represents an important virus defense strategy against host immune responses. Exposure to PRRSV did not induce DC maturation (Bordet et al., 2018; Li and Mateu, 2021). PRRSV can infect monocyte-derived DCs (Mo-DCs), resulting in reduced expression levels of major histocompatibility complex (MHC) class I (MHC-I), MHC class II (MHC-II), CD14, and CD11b/c, thus impairing their normal antigen presentation ability (Wang et al., 2007).

Other studies demonstrated that PRRSV could disrupt the swine leukocyte antigen class I (SLA-I) antigen presentation pathway. SLA-I, which is the MHC-I antigen in pigs, consists of the SLA-I heavy chain, β_2_-microglobulin (β2M), and a variable peptide. The SLA-I antigen presentation pathway, responsible for displaying peptide fragments for T-cell recognition, plays a critical role in initiating the host antiviral immune response. Previous studies showed that PRRSV nsp1α, nsp2TF, nsp4, and GP3 could each downregulate SLA-I expression on the cell surface (Du et al., 2015; Cao et al., 2016). Nsp1α mediates proteasomal degradation of the SLA-I heavy chain (Du et al., 2015). The reduction in SLA-I expression induced by nsp2TF involves the last 68 aa of the nsp2TF TF domain. Nsp4 reduces the SLA-I expression on the cell surface *via* binding to the SLA-I promoter to inhibit *B2M* transcription (Tao et al., 2018). Thus, disruption of the SLA-I antigen presentation pathway is employed by PRRSV as a strategy for modulating the host immune response.

### 3.2. Induction of regulatory T cells (Tregs)

Regulatory T cells (Tregs), the inhibitory Th lymphocytes, are responsible for modulating the immune response and maintaining homeostasis. The induction of Tregs is one of the mechanisms used by viruses such as HIV and hepatitis C virus to induce immunosuppression (Li et al., 2008). It was found that PRRSV-infected DCs significantly increased the amount of Foxp3^+^CD25^+^ T cells, which could play a role in delaying cellular immune responses in PPRSV-infected pigs during early infection (Silva-Campa et al., 2009, 2012). The recombinant PRRSV N protein was shown to play an important role in the induction of Tregs (Wongyanin et al., 2012). Further research indicated that PRRSV can induce Tregs in the lungs and tracheobronchial lymph nodes of infected pigs (Nedumpun et al., 2018).

Overall, the mechanisms by which PRRSV infection modulates host adaptive immune responses, including antigen presentation, antibody responses, T cells, and B cells, are relatively less well-characterized. Thus, more in-depth studies on these systems are needed.

## 4. Activation of immunosuppressive regulatory pathways

Interleukin-10, a pleiotropic cytokine with immuno-modulatory functions, can be produced from both the innate and the adaptive immune response, including T and B cells, DCs, and monocytes/macrophages. As a potent immunosuppressive cytokine, IL-10 has been called the “macrophage deactivation factor” because of its inhibitory effect on a variety of cytokines, such as IL-1, TNF-α, IL-4, IL-3, and granulocyte–monocyte colony-stimulating factor (GM-CSF), as well as on surface molecules, such as MHC-II proteins and co-stimulatory molecules. IL-10 is also known to have potent inhibitory effects on both antigen-presenting cell maturation and T-cell activation (Moore et al., 2001). IL-10 was reported to be responsible for the induction of PRRSV-specific Tregs and monocyte derived DCs (MoDCs) (Wongyanin et al., 2012). It has been reported that IL-10 is used by many viruses to restrict the host immune response, thus allowing the establishment of a persistent infection (Redpath et al., 2001). Indeed, monocyte treatment with IL-10 was correlated with increased susceptibility to PRRSV-1 infection (Singleton et al., 2018). Previous *in vitro* and *in vivo* studies demonstrated that PRRSV infection induces IL-10 production through the NF-κB and p38 MAPK signaling pathways (Suradhat and Thanawongnuwech, 2003; Suradhat et al., 2003; Hou et al., 2012; Song et al., 2013). PRRSV GP5 and N protein were found to be responsible for IL-10 production (Hou et al., 2012; Wongyanin et al., 2012). However, the induction of IL-10, along with several other cytokines seems to be strain-dependent (Subramaniam et al., 2011).

Notably, the immunosuppressive effect of PRRSV is not associated with just IL-10. Intracellular suppressor of cytokine signaling (SOCS) proteins are crucial intracellular regulators of innate and adaptive immunity; they are involved in the negative regulation of the JAK/STAT and TLR signaling cascades, DC activation, T-cell differentiation, and Th-cell regulation. Our lab found that PRRSV induces SOCS1 and SOCS3 production in PAMs, monkey-derived Marc-145 cells, and porcine-derived CRL2843-CD163 cells (Luo et al., 2020, 2021). SOCS1 is produced *via* the p38/AP-1 and JNK/AP-1 signaling pathways rather than *via* the classical type I IFN signaling pathways and it inhibits the expressions of IFN-β and ISGs, thereby markedly enhancing the level of PRRSV replication (Luo et al., 2020).

Interleukin-1Ra, a member of the IL-1 family, can competitively bind to the IL-1 receptor to block intracellular IL-1 signaling pathways. IL-1Ra modulates the production of many cytokines, such as IL-1, TNF-α, and type I IFN (Marsh et al., 1994; Arend et al., 1998; Arend, 2002; Banda et al., 2005). The cells primarily infected by PRRSV are members of the myeloid cell population, which are responsible for IL-1Ra production (Arend, 2002). It has been reported that PRRSV, both *in vitro* and *in vivo*, can induce IL-1Ra production, which plays an important role in the reduction of pro-inflammatory cytokine and type I IFN production during the early phase of PRRSV infection, as well as in T-lymphocyte differentiation and proliferation (Nedumpun et al., 2017).

## 5. Immune evasion *via* micro (mi)RNA regulation

Micro (mi)RNAs are evolutionarily conserved small non-coding RNAs of approximately 22 nucleotides in length; they act as critical posttranscriptional modulators of gene expression and participate in modulating immune responses (O’Connell et al., 2010). Viruses can utilize host microRNAs to modulate host immunity in ways that enable viral replication (Cullen, 2013). For example, PRRSV infection can upregulate miR-30c by activating the NF-κB signaling pathway to target *JAK1* and consequently inhibit the type I IFN signaling pathway, thereby promoting PRRSV infection. Importantly, miR-30c was found to increase following PRRSV infection *in vivo*, and a positive correlation between miR-30c production and PRRSV viral load was observed (Zhang et al., 2016). Another mechanism by which miR-30c inhibits type I IFN signaling is its targeting of the 3’ UTR region of *IFNAR2*, which results in the downregulation of IFNAR2 (Liu et al., 2018). MYH9 can be used as a functional receptor by PRRSV to interact with viral protein, and let-7f-5p was demonstrated to be a key regulator of MYH9, acting by targeting the 3’ UTR of both pig and monkey MYH9. Notably, PRRSV infection downregulates let-7f-5p production to promote the expression of MYH9 and facilitate viral replication (Li et al., 2016). MiR-24-3p and miR-22 promote PRRSV replication by directly targeting the 3’UTR of heme oxygenase-1 (HO-1), a critical cytoprotective enzyme shown to have antiviral properties, thus downregulating its expression (Xiao et al., 2015; Xiao S. et al., 2016). Latest studies have shown that PRRSV induces the upregulation of HOXA3, which can negatively regulate HO-1 gene transcription, thereby weakening the interaction between HO-1 and IRF3 for inhibiting the type I IFN response (Feng et al., 2022).

## 6. PRRSV modulates apoptosis

Apoptosis is a type of programmed cell death that is characterized by morphological changes including cell shrinkage, nuclear condensation, and plasma membrane blebbing (Zamaraev et al., 2017). It can be induced *via* two major pathways, the extrinsic and intrinsic pathways, both of which are regulated by caspases (Tait and Green, 2010). Apoptosis plays an important role in the development and maintenance of homeostasis, as well as in host defense following viral infection (Orzalli and Kagan, 2017). Apoptosis can limit virus replication by eliminating infected cells. However, viruses can also manipulate the apoptotic machinery to their advantage. Some viruses inhibit apoptosis to prevent cell death, thus increasing the production of progeny viruses, and some other viruses induce apoptosis to enhance the transmission of progeny viruses by avoiding the immune response owing to the non-inflammatory nature of apoptosis (Arora et al., 2016). It has been well-documented that PRRSV induces apoptosis *in vitro* and *in vivo*, both directly in infected cells and indirectly in bystander cells (Suárez et al., 1996; Sur et al., 1997; Labarque et al., 2003; Karniychuk et al., 2011; Gómez-Laguna et al., 2013; Novakovic et al., 2017). Later research found that the HP-PRRSV strain induces apoptosis in the bone marrow cells of infected piglets (Wang et al., 2016). And apoptosis also occurred in the thymus of piglets infected with HP-PRRSV (Wang et al., 2015). It was postulated that proapoptotic signals are delivered to thymocytes by thymic macrophages to affect thymic functions (Wang et al., 2020). The regulatory roles of PRRSV-encoded products in apoptosis have also been studied. The involvement of GP5 with the pro-apoptotic functions of PRRSV has been extensively demonstrated (Suárez et al., 1996; Fernández et al., 2002; Lee et al., 2004; Mu et al., 2015). PRRSV E protein was found to interact with mitochondrial proteins and induce apoptosis by inhibiting ATP production (Pujhari and Zakhartchouk, 2016). Nsp4 and nsp10 were also shown to be apoptosis inducers (Ma et al., 2013; Yuan et al., 2016). However, opposing views that PRRSV instead inhibits apoptosis have been reported as well (Labarque et al., 2003). It has been hypothesized that inhibition of apoptosis takes place in the early phases of infection, probably as a mechanism to avoid abortive replication by a premature death of the macrophage (Costers et al., 2008). Therefore, the mechanisms of the role of apoptosis in PRRSV infection still need to be thoroughly elaborated.

## 7. Modulating host translation

PRRSV infection, *via* an nsp2-related mechanism, induces host translation shutoff through the phosphorylation of eIF2α and the attenuation of the mTOR signaling pathway (Li et al., 2018). In addition to interfering with cellular protein production by targeting translation factors, PRRSV also does so by targeting mRNA trafficking. Because nucleocytoplasmic trafficking of molecules is an important process for many cellular functions of the host, numerous viruses target it for their own benefit. Nucleoporin 62 (Nup62) is one of the major nucleoporins located in the core of the nuclear pore complex (NPC), which is the gateway for nucleocytoplasmic trafficking. PRRSV blocks nucleocytoplasmic trafficking, thereby blocking host mRNA nuclear export, leading to inhibited antiviral protein production and thereby promoting virus growth. The responsible viral factor was identified as nsp1β, whose interaction with Nup62 disintegrated NPC (Han et al., 2017; Ke et al., 2019).

## 8. Concluding remarks

This review gives a brief overview of the various mechanisms by which PRRSV has been found to evade the host. PRRSV interferes with innate immune responses in multiple ways, such as avoiding host recognition, impairing signaling pathways that lead to the inhibition of type I IFNs, ISGs, and pro-inflammatory genes, and dysregulating NK-cell function. The suboptimal innate immune responses subsequently exacerbate the ineffectiveness of the adaptive immune response to PRRSV, which is directly targeted by the virus *via* disrupting antigen presentation, impairing DC and NK-cell functions, and inducing Tregs. In addition to targeting the host innate and adaptive immune responses, PRRSV also employs other mechanisms to escape elimination by the host, such as regulating miRNAs, apoptosis, and host translation. Although much progress has been made in all aspects of PRRSV research since the discovery of this virus, an effective means for PRRS prevention and control is still lacking. The most important strategy for controlling and eliminating viral diseases is vaccination. We are still far from reaching this goal for PRRS, possibly because understanding of PRRSV infection and immune evasion mechanisms is still incomplete. The ability of PRRSV to escape from its host has not been studied as widely compared with this ability of some important human pathogens, such as HIV. Thus, studies aimed at deciphering PRRSV host evasion mechanisms are urgently needed to guide the development of vaccines and antivirals against PRRSV.

## Author contributions

GZ and X-XC: conceptualization and review. X-XC: original draft. X-XC and JW: visualization. SQ, RL, and XL: providing guidance and modifying the review. All authors contributed to the article and approved the submitted version.

## Footnotes

1
https://talk.ictvonline.org
